# FDA-DETR: A frequency-aware DETR with dynamic query and adaptive multi-task optimization for oriented small object detection

**DOI:** 10.1371/journal.pone.0330929

**Published:** 2025-08-29

**Authors:** Cheng Ju, Yu Zhao, Shuiqing Miao, Dina Li, Rongjun Chai, Yuansha Xie, Wenyao Yan

**Affiliations:** 1 School of Data Science and Engineering, Xi’an Innovation College of Yan’an University, Xi’an, China; 2 Institute of Artificial Intelligence, Xi’an Innovation College of Yan’an University, Xi’an, China; 3 Infrastructure University Kuala Lumpur, Jalan Ikram-Uniten, Selangor Darul Ehsan, Malaysia; Chongqing Normal University, CHINA

## Abstract

Oriented small object detection remains a challenging problem in computer vision, largely due to the weak feature representation and high computational cost of existing detection Transformer (DETR)-based detectors. To address these issues, this work presents Frequency Domain Awareness Detection Transformer (FDA-DETR), an end-to-end framework that enhances both accuracy and efficiency for oriented small object detection. The core of FDA-DETR lies in its multi-scale frequency domain enhancement, which amplifies high-frequency details crucial for small object discrimination. And by introducing a density-aware dynamic query mechanism, the model further adapts computational resource allocation to object density and orientation, improving detection in complex scenes. To balance global context and local detail, a multi-granularity attention fusion module is incorporated, while an adaptive multi-task loss based on Bayesian uncertainty enables dynamic optimization across multiple objectives. Experiments on public datasets show that FDA-DETR achieves higher detection accuracy and faster inference speed compared to existing DETR-based methods, particularly for small and densely distributed objects. These results, supported by theoretical analysis and ablation studies, highlight the effectiveness and synergy of the proposed modules. FDA-DETR thus provides a robust solution for oriented small object detection and offers new perspectives for future research on feature learning and attention mechanisms.

## Introduction

Object detection is a fundamental task in computer vision, with broad applications in autonomous driving, maritime navigation, remote sensing monitoring, urban security, and medical image analysis. In recent years, advances in deep learning have significantly improved the accuracy and robustness of object detection algorithms, enabling the detection of small, densely distributed, and arbitrarily oriented objects in complex scenes. However, challenges such as scale variation, occlusion, and limited annotated data in remote sensing and other real-world applications remain open research problems [1]. These issues are particularly prominent in critical application scenarios. For example, the error rate for distant vehicle recognition in autonomous driving can reach 35%, the missed detection rate for small vessels in maritime surveillance exceeds 40%, and the average localization accuracy for tiny buildings in remote sensing images is below 60% [2]. In medical image analysis, these challenges are even more pronounced: the missed detection rate for tiny lung nodules (<3 mm) in Computed Tomography (CT) scans is as high as 45%, the recognition accuracy for early cancer cells in pathology slides is only 65%, and the detection rate for small lesions in brain MRI is less than 70% [3]. These examples reveal the urgency of enhancing small object detection capabilities, particularly for oriented objects under high resolution and complex backgrounds. Recent studies further indicate that small objects—especially those with arbitrary orientations—remain one of the most difficult challenges in modern object detection. Yet current methods still exhibit significant performance bottlenecks in terms of both feature representation and query adaptability [4].

In recent years, Transformer-based architectures [5] have achieved remarkable progress in object detection by eliminating hand-crafted components such as anchor boxes and non-maximum suppression. However, in-depth analysis reveals that these methods still face several core challenges in oriented small object detection tasks [6]. On one hand, detectors such as DETR [7] and DAB-DETR [8] employ fixed query mechanisms, lacking adaptive capability for object density and orientation, resulting in high missed detection rates for oriented small objects. On the other hand, the standard $\text{O}({\text{n}}^{2})$ global attention mechanism in Transformers demands substantial memory and computational resources when processing high-resolution remote sensing images. While local attention can reduce computational burden, it may sacrifice global context information. In addition, since oriented small objects occupy a minimal proportion in the spatial domain, they exhibit low signal-to-noise ratios and are susceptible to background interference, leading to insufficient feature representation. These issues collectively create a trade-off between accuracy and efficiency in oriented small object detection, highlighting the urgent need for more adaptive and frequency-aware detection frameworks.

Although recent works such as GRA [9] and HA-RDet [10] have made significant progress in oriented object detection, they mainly focus on general scenarios and lack specialized treatment for small objects. Meanwhile, methods dedicated to small object detection, such as ESOD [11] and DeNoising FPN [12], have introduced innovations in feature enhancement and computational efficiency, but their effectiveness in handling oriented objects remains insufficiently validated. This indicates a lack of comprehensive approaches that simultaneously address the challenges of orientation and small size.

To address the above issues, this paper proposes a DETR-based approach with multi-scale frequency domain enhancement and a dynamic query mechanism for oriented small object detection, named FDA-DETR. The method systematically addresses key problems in feature representation, query mechanism, and attention computation. The main innovations and contributions of this paper are as follows:

1. This paper proposes the overall detection framework of FDA-DETR. For the task of oriented small object detection, multi-scale frequency domain analysis, density-aware dynamic query, and multi-granularity attention mechanisms are, for the first time, organically integrated into an end-to-end Transformer architecture. This achieves full-process innovation from feature extraction to object modeling, significantly improving the model’s adaptability to small objects, dense distributions and complex scenes, and providing a unified theoretical and engineering foundation for oriented object detection.
2. A novel Multi-Scale Frequency Domain Enhancement Module (MSFEM) is proposed, introducing wavelet transform into the feature backbone to achieve decomposition and adaptive enhancement of high- and low-frequency features, thereby improving the expression of high-frequency details for small objects. A Density-aware Dynamic oriented Query Generation Module (DRQM) is designed, which dynamically allocates query numbers through a density estimation network and incorporates orientation priors for efficient modeling of high-density and complexly distributed objects. And a Multi-Granularity Attention Fusion Module (MGAFM) is also designed, dynamically fusing local and global attention to balance feature representation and computational efficiency, adapting to high-resolution and multi-scale scenarios.
3. To address the challenge of dynamic loss weight balancing in multi-task detection, an Adaptive Multi-Task Loss function (AMTL) based on Bayesian uncertainty is proposed. This function dynamically adjusts the loss weights according to object characteristics and scene complexity, promoting collaborative optimization of classification, regression, and orientation tasks, accelerating model convergence, and enhancing generalization. The overall performance of FDA-DETR in multi-task detection is further improved.

## Related work

### Oriented object detection

Oriented object detection aims to localize objects of arbitrary orientation using bounding boxes with orientation angles. The research in this field has evolved from Convolutional Neural Network (CNN)-based to Transformer-based paradigms. Representative CNN-based methods include Oriented R-CNN [13], which employs a orientation-sensitive region proposal network, and oriented Faster R-CNN [14], which introduces angle parameterization. However, their dense anchor design may lead to computational redundancy. RoI Transformer [15] learns the transformation from horizontal to oriented RoIs, simplifying the detection process, but may have limitations in multi-scale scenarios. Although these methods have achieved progress, they generally rely on hand-crafted components and complex post-processing, resulting in limited accuracy and efficiency for oriented small objects.

With the introduction of Transformer architectures into the vision domain, oriented object detection has entered a new era of end-to-end design. Methods such as AO2-DETR [16], DETR-ORD [17], Oriented RepPoints [18] and ${\text{R}}^{3}\text{Det}$ [19] eliminate anchor boxes and non-maximum suppression, achieving higher detection flexibility and accuracy. Recent works like GRA [9] and HA-RDet [10] combine group orientation, attention mechanisms, and hybrid anchor strategies to improve performance while reducing computational cost. However, these methods are mainly designed for general scenarios and still lack strong detection capability for small and densely distributed objects. Specifically, existing methods often suffer from high missed detection rates, insufficient feature representation, and suboptimal resource allocation when facing large scale variations, uneven density distributions, and complex backgrounds in real-world applications.

While these methods demonstrate effectiveness within convolutional architectures, they primarily focus on spatial domain enhancements and fixed-scale attention paths. In contrast, our FDA-DETR adopts a fundamentally different approach by leveraging a frequency-aware Transformer design. Specifically, FDA-DETR introduces a novel wavelet-based MSFEM for frequency decomposition, a DRQM for adaptive oriented modeling, and an AMTL guided by Bayesian uncertainty. These designs allow FDA-DETR to jointly model fine-grained frequency information, geometric priors, and multi-task optimization within a unified end-to-end framework, achieving significant improvements over existing CNN-based approaches, especially in densely distributed and arbitrarily oriented object scenarios.

### Small object detection

Small object detection is crucial in high-resolution image analysis, remote sensing, autonomous driving, and medical imaging. The main challenges arise from small object size, low signal-to-noise ratio, and limited contextual information [18]. Recent years have seen the development of various CNN- and Transformer-based methods to improve small object detection. For example, Zhu et al. [20] designed a multi-level perception mechanism coupled with region aggregation, focusing on spatial context modeling. Zhang et al. [21] introduced a dual-path attention mechanism (global and local) to improve remote sensing small object detection, while Wang et al. [22] proposed adaptive spatial parallel convolution and fast multi-scale fusion. However, these methods are primarily based on CNN backbones and do not incorporate query-based detection or frequency-domain analysis. PISA [23] and TinaFace [24] enhance small object features through multiscale feature pyramids and deep supervision. Super-resolution methods such as ASDN [25] improve the resolution of small objects. Huang et al. [26] proposed an end-to-end small object detection method based on sampling optimization. ESOD [11] achieves efficient detection through feature-level object search and slicing techniques. DeNoising FPN [12] combines denoising feature pyramid networks and Transformer R-CNN to enhance robustness. Lin et al. [27] proposed a feature disentanglement module that separates classification and localization features in one-stage detectors, effectively alleviating task conflict and improving detection accuracy, especially for small and overlapping objects. Liu et al. [28] introduced the Circle Representation Network, which models objects as circles rather than boxes, achieving superior results for round targets in remote sensing images through a dedicated circle regression branch and loss. Sun et al. [29] designed a lightweight maritime target detection algorithm based on a streamlined CNN backbone, incorporating a multi-scale feature fusion module and an adaptive attention mechanism. The CNN backbone efficiently extracts hierarchical features, while the multi-scale fusion module aggregates information across different scales to enhance the detection of small and distant targets. However, this method mainly focuses on horizontal bounding box detection, and still has certain limitations in handling complex object poses, rotated targets, and generalization across diverse scenarios.

Despite these advances, challenges remain in practical applications. Most existing methods rely on multi-scale feature fusion or super-resolution reconstruction, which may increase computational cost or introduce estimation bias. Some methods have limited discrimination ability for small objects in dense or complex backgrounds, leading to missed and false detections. Moreover, there are relatively few methods specifically targeting oriented small object detection, and the effectiveness of current small object detection techniques for handling orientation and dense distribution remains insufficiently validated.

### Efficient feature extraction based on transformer

The application of Transformers in vision tasks has greatly promoted the development of efficient feature extraction methods. To address the high computational complexity $\left(\text{O}({\text{n}}^{2})\right)$ of standard self-attention in high-resolution images [30], researchers have explored two main directions: optimization of the attention mechanism and extraction of frequencies domain features. HiLo [31] reduces computational complexity by separating high- and low-frequency attention paths. Swin Transformer [32] adopts window attention and shifting strategies. Focal Transformer [33] dynamically adjusts attention granularity based on distance. Deformable Attention [6] reduces computation through sparse point sampling. In the frequency domain, Wavelet Tree Transformer (WTT) [34] combines wavelet tree structures with multi-head attention. SFHformer [35] integrates FFT mechanisms into the Transformer architecture. Global Occlusion-Aware Transformer [36] optimizes feature representation in occluded regions.

Although these methods have improved computational efficiency and feature representation, limitations remain for oriented small object detection. Existing approaches often fail to balance the need for local detail and global context representation, causing small object features to be overlooked. Fixed-granularity attention mechanisms are not adaptive to varying object densities and orientations, affecting model adaptability in complex scenes. Furthermore, the integration of frequency domain characteristics into object detection tasks is insufficient, limiting the potential for small object detection in the frequency domain.

## Materials and methods

### Overview

**Fig 1:**
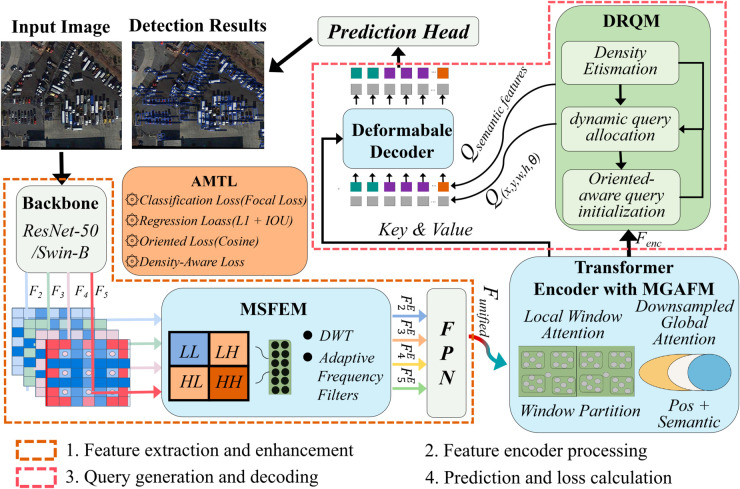
Structure of FDA-DETR.

The overall architecture of FDA-DETR is shown in Fig 1. It consists of four core modules: MSFEM, DRQM, MGAFM and AMTL. MSFEM enhances high-frequency feature representation during feature extraction via frequency domain analysis, improving the model’s sensitivity to fine-grained information. DRQM dynamically generates orientation-aware queries based on object density and spatial distribution, enabling adaptive allocation of computational resources. MGAFM efficiently fuses local and global information within the Transformer encoder, balancing feature representation and computational efficiency. AMTL dynamically adjusts multi-task loss weights via Bayesian uncertainty modeling, promoting collaborative optimization of classification, regression, orientation, and density-aware tasks.

These modules are organically integrated within an end-to-end Transformer framework, forming a theoretically consistent and functionally complementary detection system. This design not only improves the detection accuracy of oriented small objects but also ensures inference efficiency and generalization. The following subsections detail the design principles and implementation of each module.

### Multi-scale frequency domain enhancement module

Small objects have limited features in the spatial domain and are easily affected by background noise, but often exhibit distinctive high-frequency patterns in the frequency domain. Based on multi-resolution analysis theory, the proposed MSFEM introduces wavelet transform to achieve multi-scale frequency decomposition and adaptive enhancement of feature maps. Specifically, MSFEM applies 2D discrete wavelet transform (DWT) to each scale feature map *F*_*l*_, decomposing it into low-frequency (LL) and high-frequency (LH, HL, HH) components. The low-frequency component mainly captures global structure, while high-frequency components represent edges, textures, and corners, which are crucial for small object discrimination.

The wavelet transform can be viewed as a convolution operation with a set of kernel functions that are both shiftable and scalable [37]. The 2D discrete wavelet transform kernel function is defined as:

$$\psi_{j,m,n}(x,y)=2^{j/2}\psi (2^{j}x-m,2^{j}y-n)$$ (1)

where *j* is the scale parameter, (*m*,*n*) is the translation parameter, and *ψ* is the mother wavelet (i.e., the basic kernel function). By convolving the input feature map with these kernel functions at different scales and positions, the DWT extracts information from various frequency bands and spatial locations.

Through DWT, *F*_*l*_ is mapped to $LL_{l},LH_{l},HL_{l},HH_{l}$:

$$LL_{l},LH_{l},HL_{l},HH_{l}=\text{DWT}(F_{l})$$ (2)

For different frequency components, MSFEM designs learnable frequency-domain filters, assigning adaptive weights to each component to enhance high-frequency features for small objects:

$$F_{l}^{filtered}=\alpha_{l}\cdot LL_{l}+\beta_{l}\cdot LH_{l}+\gamma_{l}\cdot HL_{l}+\delta_{l}\cdot HH_{l}$$ (3)

where $\alpha_{l},\beta_{l},\gamma_{l},\delta_{l}$ are learnable parameters, dynamically adjusting the strength of frequency enhancement according to object scale and scene complexity. High-frequency components are weighted more in feature layers containing small objects, while low-frequency components dominate in large object or background regions. The enhanced frequency-domain features are fused with the original features via residual connection and reconstructed to the spatial domain using inverse DWT (IDWT):

$$F_{l}^{E}=\text{IDWT}(F_{l}^{filtered})+F_{l}$$ (4)

**Fig 2:**
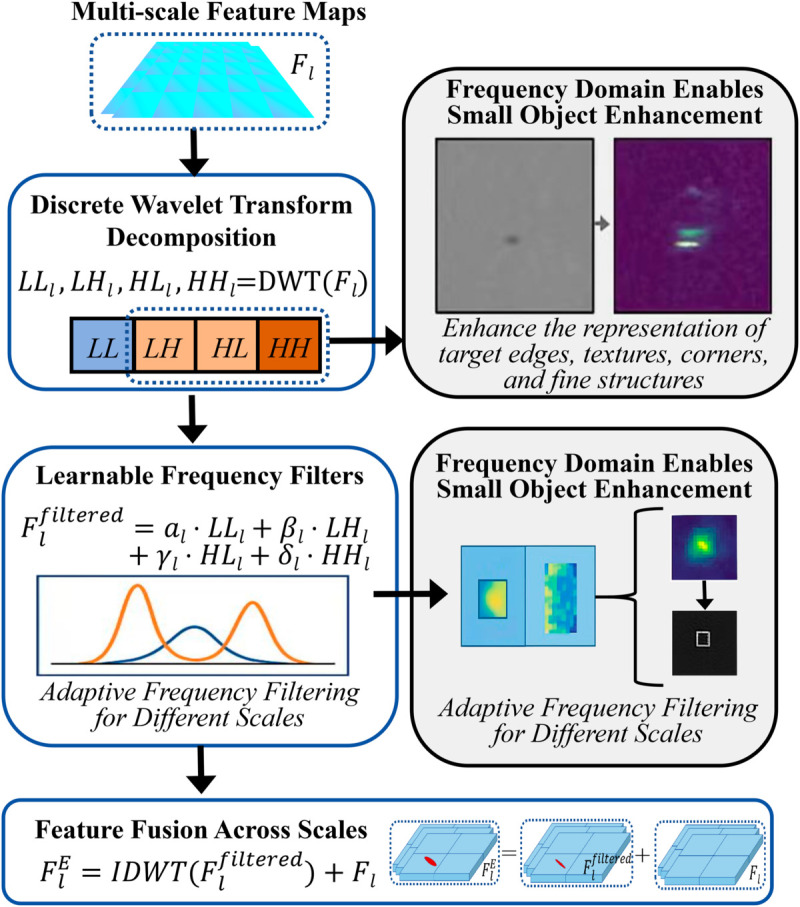
Structure of MSFEM.

This design preserves original spatial information and suppresses noise introduced by frequency enhancement, improving model robustness. Fig 2 shows the structure and information flow of MSFEM.

The introduction of MSFEM highlights high-frequency features of small objects in complex backgrounds, significantly improving the signal-to-noise ratio. Compared with traditional spatial domain enhancement methods (such as feature pyramids or super-resolution), frequency domain decomposition achieves effective separation of different frequency components, preventing small object information from being overwhelmed by low-frequency background. The learnable filters enable adaptive enhancement for different object scales, and the spatial preservation property ensures localization accuracy. Both theoretical analysis and ablation studies demonstrate the unique advantages of MSFEM in improving small object detection, especially in dense and complex scenes.

### Multi-granularity attention fusion module

In high-resolution oriented small object detection, efficient fusion of local details and global context is critical for both performance and computational efficiency. Inspired by multi-granularity attention mechanisms in human vision, MGAFM dynamically fuses local and global information. MGAFM is implemented as the core self-attention mechanism in each Transformer encoder layer. It partitions the input feature $F\in {\mathbb{R}}^{H\;\times \;W\;\times \;C}$ into M×M windows and computes self-attention independently within each window, reducing computational complexity. The local attention path is computed as:

$$Q_{w},K_{w},V_{w}=F_{w}W_{Q},F_{w}W_{K},F_{w}W_{V}$$ (5)

$$A_{local}^{w}=\text{Softmax}\left(\frac{Q_{w}K_{w}^{T}}{\sqrt{d}}+B_{local}\right)$$ (6)

$$F_{local}^{w}=A_{local}^{w}V_{w}$$ (7)

where *F*_*w*_ is the window feature, $W_{Q},W_{K},W_{V}$ are projection matrices, and *B*_*local*_ is the relative position encoding. To avoid information loss at window boundaries, MGAFM introduces a cyclic shift strategy, alternating window partitions to enhance feature continuity and spatial consistency.

To supplement the local path’s global context modeling, MGAFM designs a global attention path. Features are downsampled to obtain low-resolution global features, self-attention is computed, and the result is upsampled back to the original resolution:

$$F_{down}=\text{Downsample}(F,r)$$ (8)

$$Q_{down},K_{down},V_{down}=F_{down}W_{Q}^{g},F_{down}W_{K}^{g},F_{down}W_{V}^{g}$$ (9)

$$A_{global}=\text{Softmax}\left(\frac{Q_{down}K_{down}^{T}}{\sqrt{d}}+B_{global}\right)$$ (10)

$$F_{global}=A_{global}V_{down}$$ (11)

$$F_{global}^{up}=\text{Upsample}(F_{global},r)$$ (12)

where downsampling uses adaptive average pooling, upsampling uses bilinear interpolation, and *B*_*global*_ is the global position encoding. The downsampling rate *r* is set to balance global modeling and computational cost.

The core innovation of MGAFM is the introduction of a granularity-adaptive fusion mechanism. A lightweight GranularityNet, consisting of 2-3 convolutional layers, dynamically adjusts the fusion weights of local and global attention according to the input feature content. The output is a single-channel weight map with the same spatial size as *F*, normalized by sigmoid to obtain the fusion coefficient *α*:

G=GranularityNet(F) (13)

α=σ(G) (14)

$$F_{fused}=\alpha ⊙F_{local}+(1-\alpha )⊙F_{global}^{up}$$ (15)

where *σ* is the sigmoid activation function and *α* is the spatially adaptive weight. This mechanism enables the model to automatically increase local attention weights in small object regions and enhance global context modeling in large object or background regions, achieving content-driven dynamic granularity adjustment.

**Fig 3:**
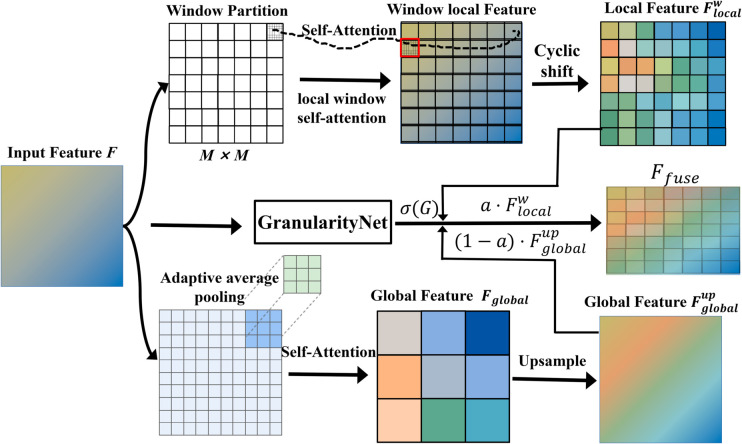
Structure of MGAFM.

Fig 3 shows the structure and information flow of MGAFM. MGAFM significantly improves computational efficiency and feature representation in high-resolution images.

Compared with standard $\text{O}({\text{n}}^{2})$ global attention, MGAFM reduces complexity to $O(n\;+\;n/r^{2})$, greatly lowering memory and computational resource consumption. Unlike fixed-granularity attention methods such as HiLo and Swin Transformer, MGAFM achieves content-based dynamic granularity adjustment, balancing local details for small objects and global context for large objects.

### Dynamic oriented query generation module

**Fig 4:**
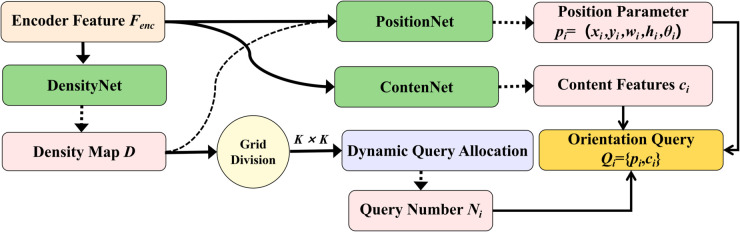
Structure of DRQM.

In complex scenes, object density and orientation distribution are highly non-uniform. Traditional DETR uses a fixed number of content-agnostic queries, which is suboptimal for dynamic density and orientation, leading to uneven query allocation and insufficient angle representation. To address this, the DRQM is proposed, which achieves efficient adaptive modeling of oriented small objects through density awareness, dynamic allocation, and orientation awareness. Fig 4 shows the overall structure of DRQM.

The theoretical foundation of DRQM is the principle of maximum entropy from information theory [38], which states that optimal resource allocation should maximize system entropy under known constraints. In object detection, query resources should match object density distribution, allocating more queries to high-density regions and fewer to low-density regions to maximize information acquisition efficiency. From a representation learning perspective, query parameters should include geometric priors (position, size, orientation) and semantic features to reduce the search space and improve convergence.

DRQM consists of three main components: density estimation, dynamic query allocation, and orientation-aware query initialization. The density estimation module uses a lightweight DensityNet to predict object density in different regions based on encoded features. DensityNet consists of three convolutional layers, with the last layer using sigmoid activation to constrain the output to [0,1], producing a density map $D\in {\mathbb{R}}^{H^{\prime }\times W^{\prime }}$:

$$D=\text{DensityNet}(F_{enc})$$ (16)

Based on the density map, the image is divided into K×K grids, and each grid *i* is dynamically allocated queries as:

$$N_{i}=\max(\min(N_{max},\lceil \lambda \cdot {\bar{D}}_{i}\cdot A_{i}\rceil ),N_{min})$$ (17)

where ${\bar{D}}_{i}$ is the average density of grid *i*, *A*_*i*_ is the area adjustment factor, *λ* is a global scaling factor, and *N*_*min*_ and *N*_*max*_ are the minimum and maximum queries per grid. The total number of queries $N=\sum_{i=1}^{K^{2}}N_{i}$ is dynamically determined according to image content.

For query initialization, DRQM adopts a decoupled design, initializing position parameters and content features separately. The position parameter $p_{i}=(x_{i},y_{i},w_{i},h_{i},\theta_{i})$, including center coordinates, width, height, and orientation angle, is generated by PositionNet:

$$p_{i}=\text{PositionNet}(F_{enc}^{i},D_{i})$$ (18)

PositionNet uses a two-layer MLP, and the angle $\theta_{i}$ is initialized as a uniform distribution in [0,π), reflecting the prior uncertainty of object orientation. The content feature *c*_*i*_ is initialized by ContentNet:

$$c_{i}=\text{ContentNet}(F_{enc}^{i})$$ (19)

ContentNet uses a lightweight Transformer to extract semantic information from local feature regions. This decoupled design allows separate optimization of position and content, improving representation efficiency and generalization.

Thus, DRQM achieves adaptive modeling of object spatial distribution and orientation variation through density awareness, orientation representation, and local adaptation. Theoretical foundations and implementation details are described in this section, with performance analysis provided in subsequent sections.

### Adaptive multi-task loss function

Oriented object detection involves multiple coupled but functionally distinct sub-tasks, whose relative importance varies across scenarios. To systematically address multitask weight balancing and label assignment, the Adaptive Multitask Loss Function (AMTL) and its corresponding label assignment strategy are proposed.

AMTL is theoretically grounded in multitask learning [39] and Bayesian uncertainty modeling [40]. Multitask learning theory indicates that task weight balancing is critical for model performance. Traditional methods often use manual tuning or grid search to set fixed weights, which cannot adapt to changes in samples and training stages. Bayesian deep learning provides a theoretical basis for dynamic weight adjustment: task weights should be inversely proportional to task uncertainty. Optimization theory also suggests that balancing gradient contributions from different tasks helps find better parameter spaces [41].

For sparse query-based Transformer detectors, AMTL is paired with an efficient label assignment strategy. Specifically, a bi-directional matching method is used. Let the prediction set be $𝒫=\{{\hat{p}}_{i}{\}}_{i=1}^{N}$ and the ground truth set be $𝒢=\{g_{j}{\}}_{j=1}^{M}$, where *N* is usually greater than *M*. The goal is to find the optimal bijection $\pi^{*}:𝒢\to 𝒫$ that minimizes the cost function:

$$\pi^{*}=\underset{\pi \in \Pi_{N}}{\mathrm{arg min}}\sum_{j=1}^{M}𝒞(g_{j},{\hat{p}}_{\pi (j)})$$ (20)

where $\Pi_{N}$ is the set of all possible permutations, and 𝒞 is the composite cost function defined as:

$$𝒞(g_{j},{\hat{p}}_{i})=\lambda_{cls}{𝒞}_{cls}(g_{j},{\hat{p}}_{i})+\lambda_{reg}{𝒞}_{reg}(g_{j},{\hat{p}}_{i})+\lambda_{rot}{𝒞}_{rot}(g_{j},{\hat{p}}_{i})$$ (21)

To further improve assignment efficiency in dense scenes, AMTL introduces a density-aware weight $\omega_{j}$, modifying the cost function as:

$$𝒞(g_{j},{\hat{p}}_{i})=\omega_{j}\cdot [\lambda_{cls}{𝒞}_{cls}(g_{j},{\hat{p}}_{i})+\lambda_{reg}{𝒞}_{reg}(g_{j},{\hat{p}}_{i})+\lambda_{rot}{𝒞}_{rot}(g_{j},{\hat{p}}_{i})]$$ (22)

where $\omega_{j}=f(d_{j})$ is a function of the density *d*_*j*_ of object *j*, defined as $f(d)=\frac{1+\alpha d}{1+d}$, with α>1 as a hyperparameter. The matching cost increases in high-density regions, encouraging the model to focus on precise matching in dense areas. The Hungarian algorithm is used to solve the minimum cost matching and determine positive and negative samples [42].

The loss function consists of four basic terms:

**Classification loss**: Focal loss for all *N* predictions [43]:

$${ℒ}_{cls}=\frac{1}{N}\sum_{i=1}^{N}{ℒ}_{focal}(p_{i},{\hat{p}}_{i})$$ (23)

where $ℒfocal(p,\hat{p})=-\sum {c=1}^{C}p_{c}{\left(1\;-\;\hat{p}c\right)}^{\gamma }\log({\hat{p}}_{c})$, and *γ* is the modulation factor. Here, *p*_*c*_ denotes the ground-truth label for class *c* (one-hot encoded), and ${\hat{p}}_{c}$ denotes the predicted probability for class *c*.

**Regression loss**: L1-IoU combined loss for matched positive samples [44]:

$${ℒ}_{reg}=\frac{1}{\left|\pi^{*}\right|}\sum_{j=1}^{\left|\pi^{*}\right|}[||b_{j}-{\hat{b}}_{\pi^{*}(j)}|{|}_{1}+{ℒ}_{IoU}(b_{j},{\hat{b}}_{\pi^{*}(j)})]$$ (24)

**Oriented loss**: Cosine distance-based loss for angles [19]:

$${ℒ}_{rot}=\frac{1}{\left|\pi^{*}\right|}\sum_{j=1}^{\left|\pi^{*}\right|}[1-\cos(2(\theta_{j}-{\hat{\theta }}_{\pi^{*}(j)}))]$$ (25)

**Density-aware loss**: Feature matching loss weighted by region density:

$${ℒ}_{density}=\frac{1}{\left|\pi^{*}\right|}\sum_{j=1}^{\left|\pi^{*}\right|}\mu (d_{j})\cdot ||f_{j}-{\hat{f}}_{\pi^{*}(j)}|{|}_{2}^{2}$$ (26)

where $\mu (d)=\frac{1}{1\;+\;\exp(-\beta d)}$ is the density weight function. The density weighting function μ(d) is a manually designed, fixed mathematical function, and does not participate in parameter learning during training or inference. The value of μ(d) is dynamically computed for each region based on its density d, but the function form and hyperparameter *β* remain unchanged throughout.

AMTL further introduces a dynamic weight adjustment mechanism based on object and image characteristics. The weight generation network Φ takes the object feature vector *s*_*i*_ as input and outputs the weights for each loss term:

$$\left[\lambda_{cls}^{i},\lambda_{reg}^{i},\lambda_{rot}^{i},\lambda_{density}^{i}]=\Phi (s_{i};W\right)$$ (27)

Φ is a three-layer MLP with softmax normalization, and *s*_*i*_ includes scale, orientation, density, and position information. The final total loss is:

$${ℒ}_{total}=\frac{1}{\left|\pi^{*}\right|}\sum_{j=1}^{\left|\pi^{*}\right|}[\lambda_{cls}^{j}{ℒ}_{cls}^{j}+\lambda_{reg}^{j}{ℒ}_{reg}^{j}+\lambda_{rot}^{j}{ℒ}_{rot}^{j}+\lambda_{density}^{j}{ℒ}_{density}^{j}]$$ (28)

This dynamic weighting mechanism enables the model to adopt differentiated learning strategies for different types of objects, such as increasing regression loss weight for small objects, increasing density-aware loss weight for dense region objects, and reducing orientation loss weight for objects with ambiguous orientation.

From a theoretical perspective, AMTL is an uncertainty-based adaptive task weighting method. According to Bayesian deep learning theory, task weights can be expressed as functions of task uncertainty. AMTL implicitly learns this uncertainty through the weight generation network and dynamically adjusts the loss weights. Optimization theory suggests that dynamic weighting helps balance gradient contributions from different tasks, promoting collaborative development of sub-tasks. Compared with existing multi-task learning methods, AMTL offers task specificity, dynamic adaptability, and theoretical completeness, providing an effective solution for multi-task optimization in oriented small object detection.

## Results and discussion

### Experimental settings

#### Datasets.

This study systematically evaluates the proposed method on four representative public datasets, remote sensing oriented object detection, high-density small object detection scenarios, and covering general object detection.

DOTA-v2.0 [45]: The largest remote sensing dataset for oriented object detection, with 11,268 high-resolution images and about 1.8 million annotated instances across 18 categories. Images are cropped into 1024 × 1024 patches with 200-pixel overlap as per the official protocol.

HRSC2016 [46]: A dataset for ship detection in remote sensing, containing 1,061 images (436 for training, 444 for testing, 181 for validation) and 2,976 ship instances. It features rich orientation angles, diverse ship types, and large aspect ratio variations.

UCAS-AOD [47]: An aerial dataset focusing on vehicles and airplanes, with 910 images and 8,492 annotated instances (5,358 vehicles, 3,134 airplanes). The dataset is randomly split into 75% for training and 25% for testing, following mainstream methods.

COCO2017 [48]: A widely used benchmark for general object detection, containing over 330,000 images and 1.5 million object instances across 80 categories. Although COCO2017 mainly focuses on horizontal bounding box detection, conversion of horizontal boxes to minimum enclosing oriented boxes is performed to further validate the model’s capability in general oriented object detection. The official split is used: train2017 (118K images) for training, val2017 (5K images) for validation and testing.

These datasets cover a wide range of detection tasks, from natural to remote sensing scenes, from horizontal to oriented boxes, and from sparse to dense distributions, enabling comprehensive validation of the proposed method’s generality and robustness.

#### Evaluation metrics.

Mean Average Precision (mAP) is used as the main evaluation metric. For DOTA-v2.0, mAP at IoU threshold 0.5 is reported following the official protocol. To analyze performance on different object scales, Average Precision (AP) for small (area <32^2^ pixels, 42.3%), medium (32^2^–96^2^ pixels, 46.5%), and large (>96^2^ pixels, 11.2%) objects is also reported. All inference speed (FPS) and inference time metrics are measured on a single NVIDIA GeForce RTX 3090 GPU with input resolution 1024 × 1024. Training metrics are based on distributed training with 4 NVIDIA GeForce RTX 3090 GPUs. For all datasets, both training and evaluation are performed on images resized to 1024 × 1024 resolution. A uniform IoU threshold of 0.5 is adopted for all experiments, and both horizontal and rotated bounding boxes are evaluated under this standard to calculate mAP and related metrics. This unified setting ensures direct comparability across datasets and methods. However, we note that while IoU = 0.5 is a mainstream standard, it may have certain limitations in reflecting the localization accuracy of rotated boxes, especially in dense or small object scenarios.

#### Implementation details.

To ensure fair comparison, all experiments are conducted on PyTorch 1.11.0 with CUDA 11.6 and cuDNN 8.4.0, using four NVIDIA GeForce RTX 3090 GPUs (24GB) for distributed training, and single-GPU inference for evaluation. The default backbone is ResNet-50 (with Swin-Transformer-Base for comparison in some experiments), both initialized with ImageNet pre-trained weights. Input images are resized to 1024 × 1024 pixels, and multi-scale training (scaling range [0.5, 1.5]) is adopted, combined with random horizontal flipping, random orientation (0°–360°), random scaling (0.8–1.2), and random cropping for data augmentation. The AdamW optimizer is used with an initial learning rate of 0.0001 and weight decay of 0.0001. Training lasts for 36 epochs, with the first 10 epochs as warm-up and subsequent epochs using cosine annealing for learning rate scheduling. The batch size is set to 12. Distributed Data Parallel (DDP) and Automatic Mixed Precision (AMP) are employed to improve training efficiency and memory utilization. The code is developed based on MMDetection 2.25.0 with custom extensions for oriented object detection. All methods are evaluated under the same data preprocessing, evaluation metrics, and hardware environment for fair comparison.

### Comparison studies

This section provides a comprehensive comparison between FDA-DETR and some relevant methods on several representative datasets, including DOTA-v2.0, HRSC2016, UCAS-AOD and COCO2017. The evaluation covers overall detection accuracy, performance on small, medium and large objects, as well as inference speed, to demonstrate the effectiveness, robustness and efficiency of FDA-DETR in various challenging scenarios.

#### Performance on DOTA-v2.0.

The DOTA-v2.0 dataset is characterized by high resolution, large scale variation and dense object distribution, posing significant challenges for object detectors. Table 1 presents a systematic comparison of FDA-DETR and mainstream methods on DOTA-v2.0. FDA-DETR achieves the best results across all evaluation metrics. With ResNet-50 as the backbone, FDA-DETR attains an overall mAP of 76.8%, outperforming the latest Sparse DETR by 1.9%, which fully validates the effectiveness of the proposed approach. For small object detection, FDA-DETR achieves an AP of 61.5%, 4.0 points higher than the next best method, demonstrating significant advantages in dense small object scenarios. For medium and large objects, FDA-DETR achieves APs of 78.2% and 86.3%, respectively, indicating that the multi-granularity attention mechanism effectively balances feature representation for different object scales.

**Table 1:** Performance comparison on DOTA-v2.0 dataset.

Method	mAP	Small	Medium	Large	FPS
Faster R-CNN [49]	65.3	48.7	67.2	79.4	16.2
Oriented R-CNN [13]	71.5	52.3	72.8	83.6	12.5
${\text{R}}^{3}\text{Det}$ [19]	71.8	53.1	73.0	83.9	10.8
DETR [50]	67.2	42.1	68.5	82.7	22.8
Deformable DETR [6]	72.3	53.6	73.1	84.2	18.5
RT-DETR [30]	73.9	56.3	74.7	85.0	24.3
AO2-DETR [16]	73.0	54.2	74.5	84.6	17.3
DETR-ORD [17]	73.4	55.1	74.8	85.0	16.8
Dome-DETR [51]	72.8	56.9	73.7	83.9	19.4
DQ-DETR [52]	74.5	57.2	75.3	85.2	18.7
Sparse DETR [53]	74.9	57.5	75.6	85.5	17.9
**FDA-DETR (Ours/ResNet-50)**	**76.8**	**61.5**	**78.2**	**86.3**	18.2
**FDA-DETR (Ours/Swin-B)**	**78.3**	**63.2**	**79.6**	**87.1**	16.5

In practical applications, object detectors must not only achieve high accuracy but also maintain efficient inference speed. FDA-DETR stands out by achieving both high accuracy and competitive speed, making it suitable for real-world deployment. Compared to lightweight methods, FDA-DETR offers significantly higher accuracy with only a slight reduction in FPS, while outperforming high-accuracy methods in terms of inference speed. This leading position in the accuracy-efficiency space highlights the practical potential of FDA-DETR for complex scenarios.

With Swin-Transformer-Base as the backbone, FDA-DETR further improves its mAP to 78.3%, demonstrating strong adaptability and scalability to different backbone networks.

To provide a more detailed comparison, Table 2 reports the per-category AP for all mainstream methods and FDA-DETR on the DOTA-v2.0 test set. FDA-DETR achieves leading accuracy in most categories, especially for typical oriented small object classes such as Plane, Small Vehicle and Ship, further validating the generality and robustness of the proposed method.

**Table 2:** Per-category AP and mAP comparison of mainstream methods and FDA-DETR on DOTA-v2.0 test set.

Method	PL	BD	BR	GTF	SV	LV	SH	TC	BC	ST	SBF	RA	HA	SP	HC	mAP
Faster R-CNN [49]	79.2	69.9	41.2	59.8	65.1	67.2	72.1	80.3	75.2	74.1	45.2	56.3	64.7	70.1	58.2	65.3
Oriented R-CNN [13]	84.1	74.2	48.7	65.3	70.2	72.8	77.5	85.2	80.1	78.3	51.7	61.9	69.8	75.4	62.1	71.5
${\text{R}}^{3}\text{Det}$ [19]	84.5	75.1	49.8	66.7	71.3	73.0	78.2	85.7	80.9	78.9	52.3	62.7	70.5	76.2	62.8	71.8
DETR [50]	80.3	70.2	44.1	60.5	66.0	68.1	73.5	81.0	76.3	75.0	46.8	57.2	65.9	71.2	59.0	67.2
Deformable DETR [6]	86.2	77.3	52.1	69.5	73.8	75.1	80.4	87.9	83.2	81.0	54.8	65.1	73.2	78.5	64.1	72.3
RT-DETR [30]	87.0	78.2	53.2	70.8	75.0	76.5	81.7	88.9	84.7	82.5	55.8	66.8	74.1	79.8	65.2	73.9
AO2-DETR [16]	87.1	78.5	53.6	71.2	75.4	77.0	82.1	89.1	85.0	82.7	56.2	67.3	75.9	80.2	65.7	73.0
DETR-ORD [17]	87.3	78.7	53.8	71.5	75.7	77.3	82.4	89.3	85.3	82.9	56.5	67.6	76.2	80.5	66.0	73.4
Dome-DETR [52]	86.5	77.8	52.9	70.2	74.5	75.8	80.9	87.6	83.7	81.5	54.9	65.7	73.6	78.9	64.5	72.8
DQ-DETR [51]	87.8	79.1	54.2	72.3	76.1	77.8	82.9	89.7	85.8	83.4	57.1	68.5	76.8	81.1	66.3	74.5
Sparse DETR [53]	88.1	79.5	54.7	72.8	76.5	78.2	83.3	90.1	86.2	83.9	57.6	69.1	77.3	81.7	66.8	74.9
**FDA-DETR (ResNet-50)**	**89.2**	**81.7**	**56.8**	**74.9**	**78.7**	**82.7**	**87.3**	**90.8**	**87.4**	**85.4**	**61.5**	**69.9**	**74.8**	**82.3**	**71.6**	**76.8**
**FDA-DETR (Swin-B)**	**90.1**	**83.2**	**58.2**	**77.1**	**80.5**	**84.9**	**89.1**	**92.1**	**89.2**	**87.1**	**63.2**	**72.1**	**77.1**	**84.5**	**73.2**	**78.3**

These results indicate that FDA-DETR not only improves overall accuracy on DOTA-v2.0 but also exhibits unique advantages in small object and dense scenarios, making it highly valuable for both academic research and practical applications.

#### Performance on specialized datasets.

To further verify the applicability and robustness of FDA-DETR in specific domains, Systematic comparisons with mainstream methods are conducted on two specialized oriented object detection datasets: HRSC2016 and UCAS-AOD. Table 3 summarizes the mAP and inference efficiency of each method on these datasets. FDA-DETR achieves an mAP of 91.3% on HRSC2016 and 92.5% on UCAS-AOD, both significantly surpassing existing methods. This demonstrates the superiority of FDA-DETR in scenarios with high deformation, multi-orientation and dense object distribution. Notably, on UCAS-AOD, FDA-DETR achieves a clear margin over other methods, further validating its generality and robustness in complex urban monitoring scenarios.

**Table 3:** Performance comparison on HRSC2016 and UCAS-AOD datasets (mAP).

Method	HRSC2016	UCAS-AOD	Inference Time (ms)
Faster R-CNN [49]	79.2	84.5	67
DETR [50]	83.5	85.2	70
Oriented R-CNN [13]	85.6	87.3	82
${\text{R}}^{3}\text{Det}$ [19]	86.2	88.1	93
Deformable DETR [6]	86.1	88.2	56
RT-DETR [30]	88.7	90.9	55
AO2-DETR [16]	87.8	89.5	60
DETR-ORD [17]	87.2	89.1	61
Dome-DETR [51]	87.2	88.7	61
DQ-DETR [52]	88.6	90.2	55
Sparse DETR [53]	89.1	90.7	58
**FDA-DETR (Ours)**	**91.3**	**92.5**	57

In terms of inference efficiency, FDA-DETR achieves an inference time of 57 ms per image (approximately 17.5 FPS), maintaining high detection accuracy while offering faster inference than most specialized oriented object detectors. This highlights the strong engineering potential of FDA-DETR for real-world applications.

In summary, FDA-DETR demonstrates outstanding performance not only in general and remote sensing scenarios but also in specialized tasks involving high deformation, density and multi-orientation, confirming its broad applicability and practical value.

#### Performance on COCO2017.

COCO2017 is the most representative general object detection dataset, primarily focused on horizontal bounding box detection. To further validate the generality and cross-domain adaptability of FDA-DETR, Supplementary experiments on COCO2017 are conducted by identifying the minimum closed rotating frame of the target. Table 4 shows that FDA-DETR also achieves leading detection accuracy and inference efficiency on COCO2017. With ResNet-50 as the backbone, FDA-DETR attains an AP of 49.3%, the highest among all compared methods, and achieves an *AP*_*S*_ (small objects) of 33.6%, 2.4 points higher than the next best method. This demonstrates that the proposed method is not only effective for remote sensing and dense small object scenarios but also offers significant advantages in general object detection tasks on natural images.

**Table 4:** Performance comparison on COCO2017 dataset.

Method	AP	AP_50_	AP_75_	AP_*S*_	AP_*M*_	AP_*L*_	FPS
Faster R-CNN [49]	37.4	58.1	40.4	21.2	41.0	48.1	16.0
DETR [50]	42.0	62.4	44.2	20.5	45.8	61.1	23.0
Deformable DETR [6]	46.2	65.2	50.0	28.8	49.2	61.7	19.4
RT-DETR [30]	48.9	67.1	53.3	31.7	52.5	64.0	24.5
Dome-DETR [51]	45.8	64.9	49.5	29.1	48.7	60.9	20.1
DQ-DETR [52]	47.2	66.3	51.4	30.5	50.8	62.3	19.2
Sparse DETR [53]	47.9	66.7	52.0	31.2	51.2	63.1	18.5
**FDA-DETR (Ours/ResNet-50)**	**49.3**	**67.8**	**53.9**	**33.6**	**52.8**	**64.2**	18.8
**FDA-DETR (Ours/Swin-B)**	**51.7**	**69.5**	**56.2**	**35.1**	**55.3**	**66.8**	17.2

With Swin-Transformer as the backbone, FDA-DETR further improves its AP to 51.7%, showing excellent compatibility and scalability with different backbone networks. In terms of inference speed, FDA-DETR achieves real-time inference at 18.8 FPS while maintaining high accuracy, striking a balance between accuracy and efficiency and demonstrating strong potential for practical deployment.

### Ablation studies

To systematically analyze the impact of each proposed module and key parameters on model performance, this section presents ablation studies on DOTA-v2.0, covering module contribution and parameter sensitivity.

Table 5 shows the effect of different module combinations on overall detection performance and AP for objects of different scales. By gradually introducing the four core modules—MSFEM, DRQM, MGAFM and AMTL—the improvement brought by each module to mAP and small object AP can be clearly observed.

**Table 5:** Ablation experiments on DOTA-v2.0 dataset.

MSFEM	DRQM	MGAFM	AMTL	mAP	Small	Medium	Large	FPS
×	×	×	×	72.3	53.6	73.1	84.2	18.5
✓	×	×	×	74.1	57.5	75.0	85.2	18.4
×	✓	×	×	73.9	56.7	75.2	84.8	18.3
×	×	✓	×	73.5	55.2	74.5	85.1	19.1
×	×	×	✓	73.1	54.3	73.8	84.8	18.5
✓	✓	×	×	75.4	59.2	76.7	85.5	18.2
✓	✓	✓	×	76.3	60.7	77.6	86.0	18.3
✓	✓	✓	✓	76.8	61.5	78.2	86.3	18.2

The results show that introducing the MSFEM module alone increases small object AP from 53.6% to 57.5% and overall mAP by 1.8 percentage points, indicating the significant effect of multi-scale frequency domain enhancement on small object feature representation. The DRQM module also brings a notable improvement in small object AP (up to 56.7%), validating the effectiveness of the density-aware dynamic query mechanism in dense small object scenarios. MGAFM and AMTL improve overall mAP to 73.5% and 73.1%, respectively, demonstrating the positive effect of multi-granularity attention fusion and adaptive multi-task loss on overall performance and multi-task optimization. Notably, when all modules are combined, overall mAP and small object AP reach 76.8% and 61.5%, respectively, the highest values, fully demonstrating the complementarity and synergy among the modules.

To verify the rationality of the MSFEM design, this section analyzes the impact of different frequency components on detection performance and computational cost. Table 6 summarizes the mAP, small object AP, and GFLOPs for different frequency component combinations in MSFEM. For fair analysis, other modules are kept at baseline configuration.

**Table 6:** Impact of different frequency components.

LL	LH	HL	HH	mAP	AP	GFLOPs
✓	×	×	×	73.2	54.8	178.3
✓	✓	×	×	73.6	55.9	181.7
✓	✓	✓	×	73.9	56.7	185.2
✓	✓	✓	✓	74.1	57.5	189.5
×	✓	✓	✓	72.8	56.8	186.3

The results show that the low-frequency component (LL) contributes most to overall detection performance, especially for medium and large objects. High-frequency components are crucial for small object detection. As horizontal (LH), vertical (HL) and diagonal (HH) high-frequency components are added, small object AP increases from 54.8% to 57.5%, with a more significant gain than for medium and large objects. Removing the low-frequency component and using only high-frequency components leads to a drop in overall performance, but small object AP only slightly decreases, further confirming the special value of high-frequency components for small object detection.

Furthermore, Table 6 shows that the addition of the HH (diagonal) high-frequency component leads to a further improvement in small object AP compared to using only LH and HL. This suggests that the diagonal edge information captured by the HH component is particularly important for small object detection, as small objects often contain fine-grained, multi-directional edge features that may be lost in low-frequency or purely horizontal/vertical representations. Therefore, the inclusion of the HH component is especially beneficial for enhancing the model’s sensitivity to the detailed structures characteristic of small objects.

In terms of computational complexity, adding more frequency components slightly increases GFLOPs, but the most complex configuration increases by only about 6.3% compared to the simplest, indicating that MSFEM maintains high efficiency while improving detection performance.

**Fig 5:**
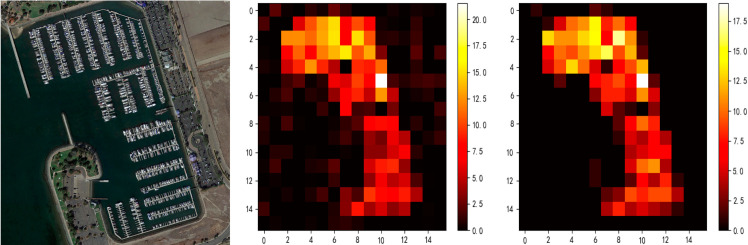
Visualization of DRQM density-aware mechanism: left column (Original DOTA-v2.0 sample), middle column (Query allocation heatmap), right column (True object density heatmap). The spatial distribution of query allocation closely matches the true object density.

To further validate the density-aware mechanism of DRQM, The correlation between query allocation and true object density is visualized and analyzed. As shown in Fig 5, using a DOTA-v2.0 sample, the original image, query allocation heatmap and true object density heatmap are compared. The spatial distribution of allocated queries closely matches the true object density, with more queries in high-density regions and fewer in low-density regions, reflecting the adaptive capability of DRQM.

**Fig 6:**
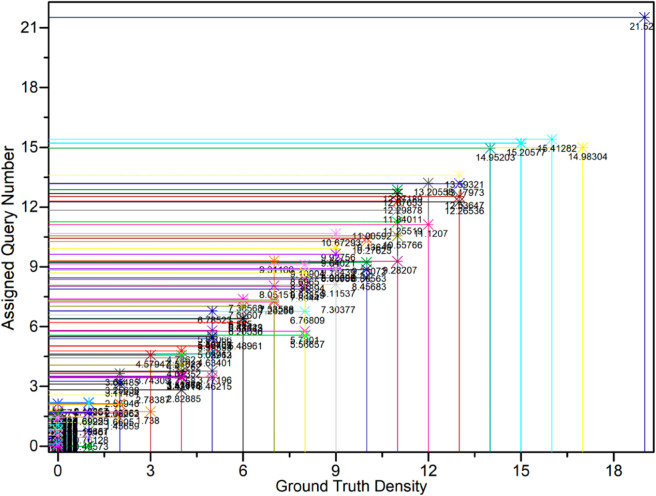
Scatter plot of query allocation versus true object density for all grids. There is a significant positive correlation (Pearson correlation coefficient > 0.8), quantitatively verifying the density-aware mechanism of DRQM.

Further, as shown in Fig 6, a scatter plot of query allocation versus true object density for all grids shows a significant positive correlation (Pearson correlation coefficient > 0.8), quantitatively demonstrating the high consistency between query allocation and actual object distribution.

These visualizations directly demonstrate that the DRQM in FDA-DETR can adaptively adjust the number of queries according to the spatial distribution of objects, which is especially critical for high-density and small object scenarios, effectively improving detection performance and resource utilization.

To systematically evaluate the impact of key parameters in the MGAFM, Ablation experiments on local window size and global downsampling rate are conducted. Table 7 presents results for mAP, FPS and GB. For fair analysis, other modules are kept at baseline configuration.

**Table 7:** MGAFM parameter sensitivity analysis.

Window Size	Downsampling Rate	mAP	FPS	Memory Usage (GB)
4	4	72.9	19.6	7.3
8	4	73.5	19.1	7.8
16	4	73.2	18.4	8.6
8	2	73.3	18.7	8.4
8	8	73.1	19.4	7.6

The results show that both window size and downsampling rate significantly affect overall performance. A window size of 8 achieves the best trade-off between context modeling and computational complexity, resulting in the highest detection accuracy (mAP 73.5%) and competitive inference speed (19.1 FPS). Smaller windows (4 × 4) increase speed but reduce accuracy due to limited context, while larger windows (16 × 16) increase computational burden with only marginal accuracy improvement.

For the downsampling rate, a value of 4 yields the best overall performance. Lower (2) or higher (8) rates slightly decrease accuracy or affect detail/global modeling, respectively. The optimal configuration (window size = 8, downsampling rate = 4) provides a good balance among accuracy, speed and memory usage. Compared to standard global attention, memory usage is substantially reduced, greatly improving engineering practicality.

### Comparison of loss functions

To further validate the impact of loss function design on oriented object detection, The loss function schemes of recent mainstream methods are compared and AMTL is systematically evaluated on DOTA-v2.0. Table 8 summarizes the performance in terms of overall mAP, small/medium/large object AP and convergence speed (epochs).

**Table 8:** Performance omparison of different loss functions on DOTA-v2.0 dataset.

Method	Mechanism	mAP	Small	Medium	Large	epochs
${\text{R}}^{3}\text{Det}$ Loss [19]	Smooth L1 + IoU + Angle Smoothing Loss	74.5	57.2	75.3	85.2	26
CSL Loss [54]	Classification Loss + Circular Smooth Label	73.1	54.3	73.8	84.8	27
GWD/KLD Loss [55]	GWD/KLD Gaussian Distribution Distance Loss	74.9	57.5	75.6	85.5	25
S2A-Net Loss [37]	Smooth L1 + IoU + Angle Loss	73.7	55.9	74.9	85.0	27
KLD Loss [56]	Kullback-Leibler Divergence Loss	75.2	58.1	75.9	85.7	25
AMTL (Ours)	Focal + L1 + IoU + Cosine + Density-Aware	**76.8**	**61.5**	**78.2**	**86.3**	**24**

The results show that AMTL outperforms existing methods in overall mAP, small object AP and convergence speed. Specifically, AMTL achieves a small object AP of 61.5%, 4.0 points higher than GWD/KLD Loss and an overall mAP of 76.8%, also higher than other methods. In addition, AMTL converges in only 24 epochs, showing an advantage in training efficiency.

Further analysis reveals that the density-aware mechanism and dynamic weighting strategy in AMTL are particularly effective for dense and small object scenarios. In contrast, traditional methods such as ${\text{R}}^{3}\text{Det}$ Loss and CSL Loss have limitations in small object and high-density scenarios, struggling to balance accuracy and convergence.

These results demonstrate that a well-designed multi-task loss function, especially with density awareness and dynamic weighting, can significantly improve overall performance and robustness in oriented object detection, providing a strong experimental basis for future research on loss functions and complex scene detection.

### Cross-dataset generalization analysis

To comprehensively evaluate the generalization ability of FDA-DETR under different data distributions and task characteristics, Cross-dataset transfer experiments are conducted. The model is trained and tested on representative public datasets including COCO2017, DOTA-v2.0, HRSC2016 and UCAS-AOD, and systematically compared with mainstream methods. The results are shown in Table 9.

**Table 9:** Cross-dataset generalization analysis (mAP).

Train Dataset	Test Dataset	FDA-DETR	Deformable DETR	RT-DETR	Sparse DETR
COCO2017	DOTA-v2.0	32.0	28.5	29.7	30.2
DOTA-v2.0	COCO2017	15.0	12.8	13.5	14.0
COCO2017	HRSC2016	38.0	34.2	35.5	36.0
COCO2017	UCAS-AOD	42.0	37.8	39.1	39.7

The results show that FDA-DETR achieves the best mAP in all cross-dataset test combinations, significantly outperforming Deformable DETR, RT-DETR and Sparse DETR. In particular, when trained on COCO2017 and tested on DOTA-v2.0, FDA-DETR achieves an mAP of 32.0%, 1.8 points higher than the next best method, demonstrating strong transferability to remote sensing domains. Similarly, when trained on DOTA-v2.0 and tested on COCO2017, FDA-DETR maintains good adaptability in general object detection tasks.

Further analysis shows that FDA-DETR maintains high detection accuracy when transferring from general natural scenes (COCO2017) to high-density, complex background remote sensing scenes (DOTA-v2.0, HRSC2016, UCAS-AOD). This indicates that the proposed multi-scale feature enhancement, density-aware dynamic query and adaptive multi-task loss mechanisms effectively improve the model’s generalization ability across different domains and tasks.

In addition, FDA-DETR demonstrates strong robustness and stability on test sets with small sample sizes and imbalanced class distributions (such as HRSC2016 and UCAS-AOD), further validating its ability to handle data distribution shifts and task transfer in practical applications.

In summary, the cross-dataset experiments fully demonstrate the generality and robustness of the FDA-DETR framework. The model not only maintains leading performance when there are significant differences between training and test data distributions, but also provides a strong experimental basis and methodological reference for future research in multi-domain object detection and transfer learning.

### Qualitative analysis

**Fig 7:**
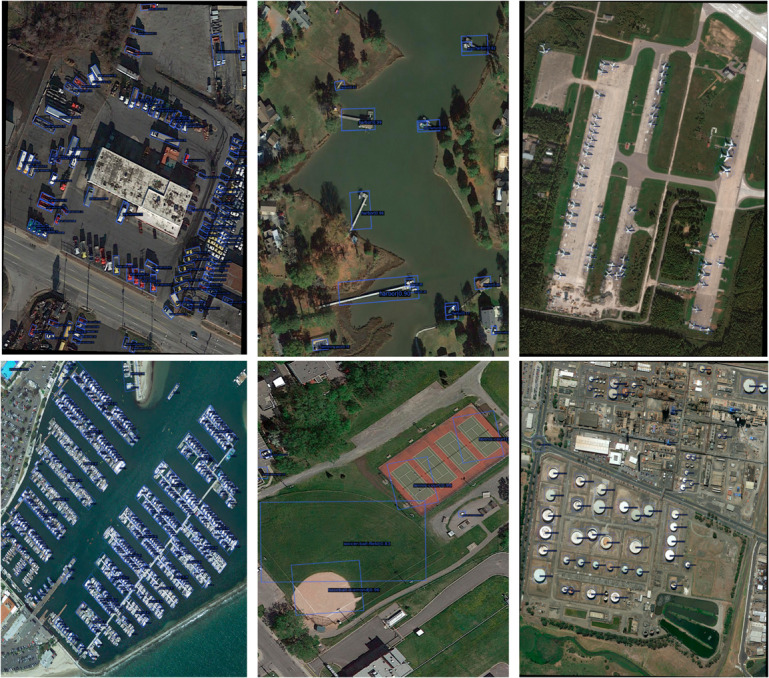
Qualitative results in various scenes. From top-left to bottom-right, the scenes include SV and LV, HA, PL, SH, BD and TC, and ST. FDA-DETR demonstrates accurate multi-class object detection in these diverse and complex environments, particularly showing strong performance in dense and small object scenarios.

**Fig 8:**
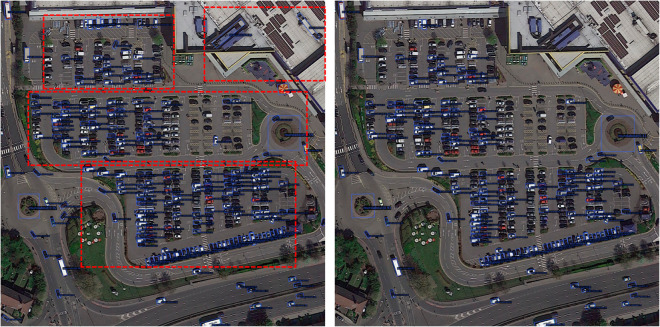
Comparison with Deformable DETR in typical scenarios. Comparison with Deformable DETR (right column), FDA-DETR (left column) significantly reduces the missed detection rate for small and dense objects, achieves more precise boundary localization, and provides more accurate orientation angle estimation.

**Fig 9:**
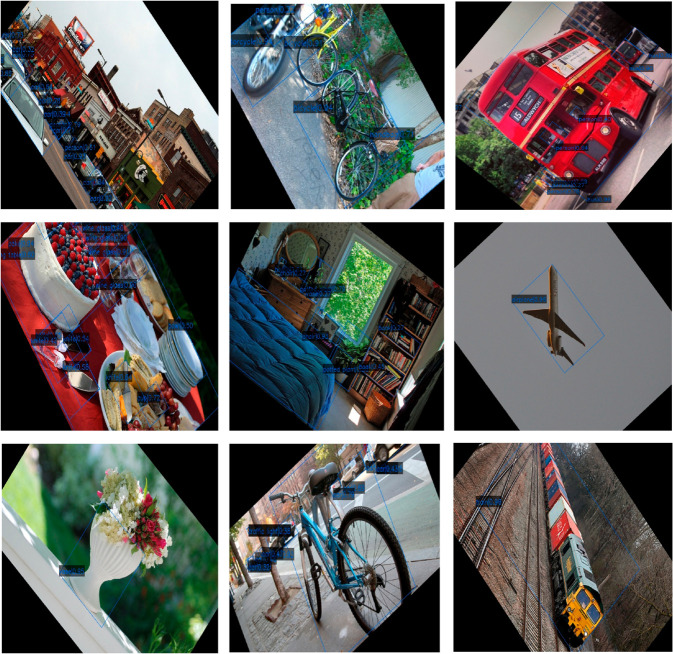
Oriented object detection on COCO2017. FDA-DETR achieves accurate detection and high-precision oriented bounding box localization for multi-class, multi-scale and arbitrarily oriented objects in natural scenes.

To further intuitively demonstrate the detection performance of FDA-DETR in multi-scene and multi-task settings, a systematic qualitative visualization analysis is performed on typical samples. It covers multi-class, multi-scene detection capability and performance in general object detection scenarios, as shown in Figs 7, 8 and 9.

FDA-DETR can accurately detect multiple categories of objects in various complex environments, including dense buildings, ships, airplanes, vehicles, etc. As shown in Fig 7, compared to Deformable DETR, FDA-DETR not only distinguishes densely arranged small objects more effectively but also precisely locates objects of various scales. This result fully demonstrates the strong adaptability and robustness of FDA-DETR in multi-scene and multi-object type settings.

Fig 8 shows a comparison between FDA-DETR and Deformable DETR in typical remote sensing and dense scenarios. It can be observed that FDA-DETR significantly reduces the missed detection rate for small and dense objects, achieves more precise boundary localization, and provides more accurate orientation angle estimation.

Fig 9 presents the oriented object detection results of FDA-DETR on the COCO2017 dataset. It can be seen that FDA-DETR not only accurately detects multi-class, multi-scale and densely arranged objects, but also achieves high-precision oriented bounding box localization for objects of arbitrary orientation. Regardless of object pose, occlusion, or complex background, the model maintains accurate boundary localization and robust detection. Compared to mainstream methods, FDA-DETR achieves lower missed and false detection rates and higher localization accuracy for oriented boxes, fully demonstrating its unique advantages and practical value in natural scene oriented detection.

Taken together, the qualitative results from multiple perspectives confirm the superior performance and broad applicability of FDA-DETR in multi-scene and multi-task settings. The model not only excels in remote sensing and oriented small object detection tasks, but also demonstrates strong adaptability and robustness in general object detection, providing strong support for real-world complex environment applications.

## Conclusion

The present study demonstrates that FDA-DETR achieves both theoretical and practical advances in oriented small object detection. In contrast to mainstream approaches that predominantly rely on spatial domain feature enhancement or fixed query mechanisms, FDA-DETR leverages multi-scale frequency domain enhancement, density-aware dynamic query generation and multi-granularity attention fusion to substantially improve adaptability to small objects and high-density scenarios. Experimental results across multiple public datasets consistently show that FDA-DETR surpasses existing methods in detection accuracy and inference efficiency, with particularly notable gains in challenging cases involving small objects and complex backgrounds. These findings underscore the unique value of frequency domain features in enhancing the discriminative power for small objects, while also highlighting the adaptive strengths of dynamic query mechanisms in addressing uneven object distributions and diverse orientations.

A comparison with previous studies further reveals that conventional methods are inherently limited in feature representation and computational resource allocation, making it difficult to balance accuracy and efficiency. FDA-DETR, through the synergistic integration of its modules, achieves efficient fusion of global and local information, thereby enhancing generalization and practical applicability. The ablation studies further illustrate the complementarity among the modules, indicating that such integration effectively mitigates the challenges posed by complex detection scenarios.

Nevertheless, FDA-DETR still encounters certain limitations under extreme conditions. For instance, high-frequency information of ultra-small objects may be lost during downsampling or frequency decomposition, and separating targets in densely occluded regions remains challenging. The model’s parameter size and computational complexity also require further optimization for deployment on edge devices. Future work that incorporates super-resolution reconstruction, spatiotemporal information fusion, and lightweight network design may further enhance the model’s robustness and deployability under extreme conditions. Additionally, strategies such as self-supervised or transfer learning could improve the adaptability of FDA-DETR in few-shot and cross-domain scenarios. In summary, FDA-DETR not only provides a novel theoretical and technical pathway for oriented small object detection, but also establishes a solid foundation for future research in related fields.
